# The effect of pregnancy pilates-assisted childbirth preparation training on urinary incontinence and birth outcomes: a randomized-controlled study

**DOI:** 10.1007/s00404-024-07653-5

**Published:** 2024-07-31

**Authors:** Gonca Buran, Serap Erim Avcı

**Affiliations:** 1 Department of Obstetrics and Gynecology Nursing, Faculty of Health Sciences, Bursa Uludag University, 16059 Nilüfer, Bursa, Turkey; 2 Gynecology and Obstetrics, Pregnancy Education and Counseling Center, Nilüfer, Bursa, Turkey

**Keywords:** Nursing, Postpartum, Pregnancy pilates, Stress incontinence, Urinary incontinence

## Abstract

**Purpose:**

To examine the effect of pregnancy pilates-assisted birth preparation training on urinary incontinence (UI) including stress urinary incontinence (SUI) and urge urinary incontinence (UUI) during pregnancy, and the postpartum period and birth outcomes.

**Method:**

In this single-center, single-blind, randomized, controlled study, 126 participants who have 28–30 weeks of gestation and nulliparous were randomly assigned to receive either the (*n* = 63) or control group (*n* = 63). The study was carried out between March and August 2022. Pregnancy pilates intervention was applied twice in a week, a total of 8 weeks to pilates group. The control group was given routine obstetric and pregnancy care. A personal data form and the Michigan Incontinence Severity Index Form (M-ISI) were used as data collection tools.

**Results:**

The mean weight gains of the experimental group during pregnancy were significantly lower than the control group. The experimental group had almost twice the rate of vaginal birth than those of the control group. The duration of labor mean score of experimental group was 5 h and 43 min less than the duration of labor of the control group (*p* < 0.001). After intervention, and postpartum period, the SUI and UUI severity of the experimental group was significantly lower than those of the control group (*p* < 0.001).

**Conclusion:**

Pilates-assisted childbirth preparation training reduced the severity of UI including SUI and UUI symptoms during pregnancy and the early postpartum. In addition, pilates-assisted childbirth preparation training contributes to decrease in weight gain during pregnancy, the increase in the vaginal birth rate, and the shortening of the duration of labor.

**Trial registration:**

NCT06185439

## Introduction

Urinary incontinence (UI) is defined as unintentional urine loss or leakage, which has such a severe social and negative hygienic influence that an undesirable situation develops. It is a common condition experienced by a significant proportion of the female population globally [1, 2]. The type of UI that women suffer from most during pregnancy and postpartum period is stress urinary incontinence (SUI) and urge urinary incontinence (UUI) [3, 4]. Stress urinary incontinence (SUI) is characterized by the involuntary loss of urine when intra-abdominal pressure increases, such as during laughing, coughing, sneezing, or exercise [3]. Urge urinary incontinence (UUI) is defined as a complaint of involuntary loss of urine associated with urgency [3, 5]. In the general female population, the common risk factors for UI include pregnancy including age at gestation, parity, maternal Body Mass Index (BMI), weight gain during pregnancy, vaginal delivery, and pelvic surgery [6, 7].

Anatomical, physiological, and hormonal changes that occur during pregnancy and minor/major traumas that occur during birth cause the development of urinary incontinence. The incidence of SUI increases with the frequency of gestational weeks [6, 7], because there is a decrease in pelvic floor muscle strength from the 20th week of pregnancy to the 6th week postpartum. In this decrease, the pelvic floor muscles may be negatively affected by the effect of both the fetus and the hormone relaxin, which increases during pregnancy [8]. While relaxin softens the connective tissue in the pelvic floor to prepare for birth, the growing uterus pushes the pelvic organs downward. Additionally, the vaginal birth also contributes decreasing the strength in the pelvic floor. Thus, the pelvic floor is exposed to constant stress and pressure. Decreased pelvic floor muscle strength causes SUI attacks to increase [8, 9].

A previous study reported that the prevalence of SUI increases from 18.6 to 67% as the pregnancy progresses [8]. A systematic review reported that while the risk of urinary incontinence is 1% in nulliparous adolescents, it increases to 42.2% in middle-aged women, and this problem increases with age. Among those with UI, 12.5–79% were reported as having stress urinary incontinence, and 15.6–41.6% were reported as having UUI. The prevalence of SUI in the postpartum period varies between 7 and 56%. Risk factors for UUI and SUI in the postpartum period are related to the mode of delivery, the number of births, and the presence of SUI during pregnancy [5, 11, 12].

Despite the anatomical and physiological changes in the pregnant body, the American College of Obstetricians and Gynecologists (ACOG) recommends moderate-intensity aerobic exercise for at least 20–30 min a day during the pregnancy period [13]. In childbirth preparation classes, nurses and midwives include exercises for pregnant women in their programs. Pilates is also included among these exercises [13–15]. Pilates exercises during pregnancy can be adapted to help women strengthen their pelvic floor muscle, manage their flexibility, and maintain joint mobility [14, 16, 17].

It is known that the most effective way to protect from SUI during pregnancy and the postpartum period is pelvic floor muscle exercise [1]. There is no strong evidence yet that exercise regimens other than pelvic floor muscle training can reduce UI in women during pregnancy and postpartum. It has been determined that the majority of different pregnancy exercise and pregnancy pilates studies focus on the relationship between birth outcomes, fear of birth, and quality of life [9, 14, 15, 18]. While the studies conducted so far have shed light on the issues faced by pregnant women, such as low quality of life, fear of birth, low back pain, BMI, and birth outcomes, there are still some gaps in the literature. It is important for nurses and midwives to provide evidence-based education, care, and consultancy services in childbirth preparation classes. For these reasons, in this randomized-controlled study, we aimed to examine the effect of pregnancy pilates-assisted childbirth preparation training on birth outcomes, urinary incontinence during pregnancy, and the postpartum period. The research questions are listed below:

- 1. Is there a different mean score of urinary incontinence between the experimental group and the control group during pregnancy?
- 2. Is there a different mean score of urinary incontinence between the experimental group and the control group in the postpartum period?
- 3. Are the BMI mean scores of women in the experimental group lower than those of women in the control group during pregnancy and postpartum?
- 4. Is there a difference in vaginal birth rates between the experimental and control groups?
- 5. Is there a difference in mean labor duration scores between the experimental and control groups?

## Materials and methods

### Design of the study

The research was an experimental study with parallel groups including single-blind randomized-controlled, experimental and control with educational intervention. This clinical trial was registered at ClinicalTrials.gov (ref. no: NCT06185439). Reporting adhered to the CONSORT extension for parallel group randomized trials [19] and the TIDieR checklist [20].

### Study setting

This study was conducted between March 2022 and August 2022 at the Gynecology and Obstetrics, Pregnancy Education and Counseling Center. In the country where the study took place, pregnancy exercises and childbirth preparation courses are not part of routine pregnancy care. Pregnancy education is provided in short periods during routine pregnancy follow-up visits. One of the main reasons for choosing this center is that there are childbirth preparation classes and pregnancy exercise rooms in this center. The other reason is that the pregnant women in the country prefers this center for routine obstetrics care.

### Study population and sample size

The sample size of the research was determined by power analysis using program the G. Power-3.1.9.2″ [21]. It was calculated that 57 women had to be included in each group to reach a 95% power at the 5% types I error level with a 0.60 (medium) impact width. Due to possible losses, 63 women were invited to each group in the study. However, a total of 122 women completed the study for reasons such as pregnancy complications and abandoning the study. Therefore, the power analysis was performed again. According to this analysis, it was determined that the study had 95% power, 5% Type I error level, and 0.60 (medium) effect width.

#### Inclusion criteria

(1) Being between the ages of 18–35 years, (2) being in the 28th–30th week of pregnancy, (3) having a singleton pregnancy, and (4) being nulliparous.

#### Exclusion criteria

(1) Having chronic constipation, (2) having a chronic cough, (3) giving birth outside of 37–42 weeks, (4) being overweight and obese, (5) having a history of any psychiatric condition, spinal cord injury, multiple sclerosis, bladder malignancy, or congenital or acquired anatomical abnormalities of the urogenital tract, and (6) having UI due to a secondary condition, such as chronic urinary tract infection.

### Randomization

To ensure randomized allocation, a computer-aided program called "random.org" was used to obtain an ordered and numbered list of random assignments to the experimental and control groups [22]. The researchers assigned participants to their respective groups based on the order of their arrival and the numbers in the randomization scheme.

### Blinding

Due to the nature of the study, it was not possible for the researchers to blind the participants. Because the assignment of the participants to the groups and the intervention were carried out by the researchers. However, the researchers were blinded to the analysis to avoid potential bias. The data entries were coded as A and B, and the analysis was carried out by a biostatistics expert, who was not involved in the study and remained unaware of the codes related to the groups. In addition, to ensure impartiality, the hospital’s maternity ward staff (doctors, nurses, and midwives) was blind to groups.

### Procedure

The pregnant women (*n* = 387) were evaluated for eligibility. There were 144 pregnant women who did not meet the inclusion criteria. Twelve pregnant women declined to participate in the study, while 105 others did not attend for various reasons. As all participants were in the same trimester of pregnancy, no further classification or stratification was necessary. This study was conducted in two groups: an experimental group (*n* = 63) that received the pregnant pilates intervention, and a control group (*n* = 63) that received only routine hospital care.

The study and data collection process were carried out in three stages. The first stage was pre-intervention, the second stage was post-intervention, and the third stage was postpartum period.

#### Pre-intervention (during assessment at registration)

During registration assessment, both the experimental and control groups completed a survey consisting of a five-question introductory information form and a 10-question Michigan Incontinence Severity Index Form.

#### Post-intervention

The control group received the usual care. Immediately after at the end of the 8-week prenatal pilates-assisted childbirth preparation training, evaluation of the incontinence score with the Michigan Incontinence Severity Index Form was applied to all of the groups, after the intervention. The follow-up of four participants in the control group was terminated due to their childbirth occurring outside the 37–42 gestational weeks.

#### Postpartum period (1 month following hospital discharge)

**Fig. 1:**
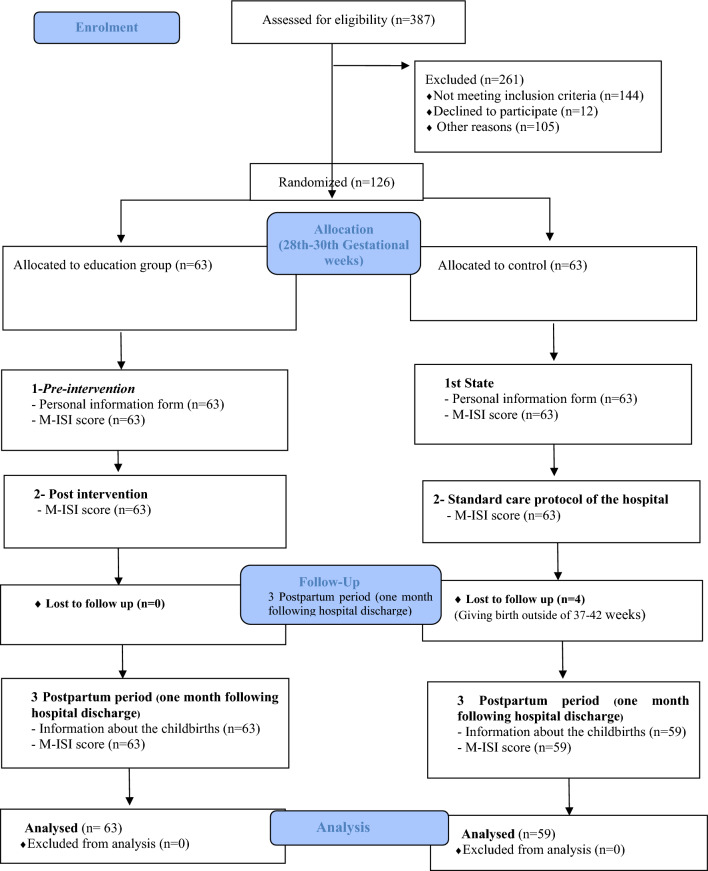
CONSORT diagram

During the postpartum period, 1 month following hospital discharge, participants were contacted by telephone and the mothers in the experimental (*n* = 63) and control groups (*n* = 59). Four participants in the control group were lost to follow-up due to giving birth outside of the 37–42-week gestational period. The participants were asked seven questions about obstetric outcomes and the M-ISI was performed to evaluate the urinary incontinence (UUI) and stress urinary incontinence (SUI) scores. All stages of the research are summarized in the Consort Diagram in Fig. 1.

### Intervention

The principal author, who has a certificate as a childbirth education course program practitioner, gave the participants who attended the experimental groups a total of 8 weeks of theoretical childbirth preparation training, including pregnancy physiology, body mechanics, changing the center of gravity during pregnancy, walking balance, breathing exercises during pregnancy and labor, the importance of exercise during pregnancy, and its contributions to the postpartum process. The second author, who has a national pregnancy pilates certificate, performed the pilates exercises. The experimental group was provided with prenatal pilates and follow-up, in addition to the usual care provided by health professionals. Training was given twice a week for 2 h a day (1 h of theory, 1 h of exercise = 45 min of pregnancy pilates, and 15 min of breathing exercises) for a total of 8 weeks by researchers.

The 8-week pregnancy pilates program comprised moderate-intensity exercise (Borg Scale 12–14), in accordance with the ACOG guidelines. The Pilates exercise program was designed with a specific structure, comprising a warm-up phase, a main exercise program, and a cooling phase. The program was conducted at the mat level, with participants engaged in exercises against gravity and resistance (in conjunction with the utilization of an exercise band and exercise ball). The initial 10 min of the 1-h exercise program comprised a series of warm-up exercises. The remainder of the program comprised a mid-load period of approximately 30 min, followed by a 5–10-min cooling period. The breathing exercises is approximately 10–15 min.

### Warm-up exercises

Warm-up exercises include Cleopatra, toy soldier, stretching the chest muscles, swinging, semi-squatting, shoulder flexion-abduction-external rotation, and cat-camel exercises. All exercise exercises were performed for 2 sets, 5 repetitions, 10 min.

### Main exercises

Main exercises are done in 2 sets on the mat, starting with 5 repetitions, increasing to 8 repetitions in the 2nd week, 10 repetitions in the 3rd and 4th weeks, and up to 15 repetitions in the 5th and 6th weeks. After the 32nd week of pregnancy, the main exercises were reduced to 2 sets of 5 repetitions and the repetition of the cooling phase exercises was increased. Main exercise program: single leg stretch, toe-to-heel, pelvic tilt on pilates ball, leg circles, side kick, clamshell, shoulder exercises, hip adductors, elbow flexors, back and chest muscle strengthening, and side semi-squat, with pilates ball. There are squatting, elbow and shoulder extensors strengthening exercises.

### The cooling phase

The cooling phase consists of 10 min of mat and stretching exercises, 2 sets and 5 repetitions each. After the 32nd week of pregnancy, cooling exercises were increased to 3 sets and 5 repetitions, depending on the evaluation of the pregnancy. Exercises performed during the cool-down phase are mermaid, falls, pectoral stretching, trunk rotation, iliotibial band stretching, and a little piece of heaven exercises.

### Measures

Research data were collected using the Michigan Incontinence Severity Index (M-ISI) and Personal Data Form.

### Personal data form

There was a total of 12 questions, with five related to sociodemographic data and seven related to birth outcomes.

### Michigan incontinence severity index (M-ISI)

The Michigan Incontinence Severity Index, Suskind et al. [23], is a scale created in 2014 to evaluate the type and severity of women’s incontinence and use of pads. The validity and reliability study of the scale in Turkey was conducted by Sargın et al. [24]. The scale consists of ten items and two sub-dimensions. It is a 10-item measure that consists of a total M-ISI domain (sum of items 1–8) and a distinct Bother domain (sum of items 9–10). The Total M-ISI score consists of three subdomains (items 1–3 for Stress Urinary Incontinence [SUI], items 4–6 for Urgency Urinary Incontinence [UUI], and items 7–8 for Pad Use [PU]). All 10 items have Likert scale response options (range 0–4), with higher values representing greater symptoms and greater bother. The Total M-ISI domain ranges from scores of 0 to 32, the Bother domain ranges from scores of 0 to 8, the SUI and UUI subdomains range from scores of 0 to 12, and the PU subdomain ranges from scores of 0 to 8. The overall domains and subdomains are scored by simply adding up their respective subdomains. The minimally important difference (MID) has been determined for the following domains/subdomains: Total M-ISI 4 points, SUI subdomain 2 points, and UUI subdomain 2 points. The overall score of the scale gives the severity of incontinence. As the score on the scale increases, it is interpreted as an increase in the level of incontinence. In the reliability study, the Cronbach Alpha coefficient was found to be 0.79 for stress incontinence and 0.86 for urge incontinence [24].

### Statistical analysis

The evaluation of the data obtained as a result of the research was analyzed with the SPSS 20.0 (SPSS Inc., Chicago, IL, USA) package program. The conformity of the data to normal distribution was evaluated with the Kolmogorov–Smirnov normality test. For descriptive statistics, number, percentage, arithmetic mean, and Standard Deviation (SD) were used. The Pearson chi-square test (expected number > 25), Yates corrected chi-square test (observed value 5 < expected number < 25), and Fisher’s exact test (expected number < 5) for categorical variables were used to test the homogeneity. Independent groups t test was used for comparisons between two groups of normally distributed quantitative variables. The Mann–Whitney *U* test was used for comparisons between two groups of quantitative variables that did not show normal distribution. Independent sample *T *tests and the Mann–Whitney *U* test of the difference between the two mean values were used to compare the Michigan Incontinence Severity Index Form (M-ISI) of the groups. It was calculated and for all tests, and < 0.05 was the standard for statistical significance.

### Ethical considerations

Ethical approval was obtained (Non-Interventional Clinical Research Ethics Committee of Bursa Uludag University (IRBN: 2022-5/20). The trial is registered at ClinicalTrials.gov (International Study Registration Number: NCT06185439). The purpose of the research was explained to the participants, they were informed that the information obtained would not be used outside the research and that the confidentiality of their personal information would be protected, and their verbal and written informed consent was obtained from all individual participants included in the study. The Principles of the Declaration of Helsinki were complied with at every stage of the research.

## Results

### Descriptive statistics of the sociodemographic characteristics of the groups

The characteristics of the pregnant women in the experimental and control groups are given in Table 1. The findings showed that the age, gestational week, Body Mass Index (BMI), educational status, employment status, and income status of the participants in the control group and the control group were not statistically significant (*p* > 0.05). They were similar and had a homogeneous structure (Table 1).

**Table 1:** Characteristic information of the experimental and control groups

Characteristics^a^	Experimental (*n*=63) Mean ±SD	Control (*n*=63)Mean ±SD	*t*^a^	*p*
Age	29.50 ± 2.57	29.09 ± 4.58	0.622	0.535
Gestational week	29.14 ± 0.80	29.01 ± 0.81	0.883	0.379
BMI (kg/m^2^)				
Pre-pregnancy	22,15 ± 2.30	21,79 ± 2.38	0.875	0.383

	*n *(%)	*n *(%)	*χ*2	*P*
Educational status				
High school	8 (12.7)	10 (15.9)	0.604	0.739
University	46 (73)	42 (66.7)		
Postgraduate	9 (14.3)	11 (17.5)		
Employment status				
Yes	44 (69.8)	51 (81)	2.096	0.148
No	19 (30.2)	12 (19)		
Income status				
Income=expenditure	35 (55.6)	45 (71.4)	3.424	0.64
Income>expenditure	18 (28.6)	28 (44.41)		

^a^Pearson’s chi-square test*, *^b^*t* test

### Information about the childbirth outcomes of the experimental and control groups

Information about the childbirth outcomes of the experimental and control groups is given in Table 2. Gestational week at birth in the experimental groups was 39.59 ± 0.58 weeks and 39.32 ± 0.97 weeks for the control group, which was not significantly different (*t* = 1.612, *p* = 0.611). The BMI of the participants in the postpartum period experimental group (21.85 ± 2.26) was lower than in the control group (22.73 ± 2.11, *t* = 1.612, *p* = 0.031). The total vaginal birth rate of the participants in the pregnancy pilates intervention group was 80.6%. Of these, 50% underwent a spontaneous vaginal birth (SVD), while 30.6% underwent a vaginal delivery under epidural analgesia (VD EA). A total of 19.4% of the women in the experimental group underwent a cesarean section. The corresponding rate in the control group was 54.2% (25.4% SVD and 28.8% VD EA). The total cesarean section rate of the participants in the pregnancy pilates intervention group was 19.4%. Of these, 14.5% underwent a cesarean section under spinal anesthesia (CS SA) and 4.8% underwent a cesarean section under general anesthesia (CS GA). In the control group, 22% of women underwent a cesarean section under spinal anesthesia (CS SA) and 23.7% underwent a cesarean section under general anesthesia (CS GA), resulting in a total of 45.7% undergoing a cesarean delivery. A statistically significant difference was identified between the experimental and control groups in terms of mode of delivery (*χ*^2^ = 13.455; *p* = 0.003). All of the mothers in the experimental group and the control group had episiotomy (*p* > 0.05). When the duration of labor of the participants who gave birth vaginally was compared, it was 9.46 ± 2.58 (hour ± minute) in the experimental group and 15.29 ± 1.77 (hour ± minute) in the control group. A statistically significant difference was determined between the groups in terms of the duration of labor (*t* = 676.5; *p* < 0.001, Table 2).

**Table 2:** Childbirths information of the experimental and control groups

Childbirths information Mode of delivery^a^	Experimental (*n*=63) *n* (%)	Control (*n*=59) *n* (%)	*χ*2	*p*^a^
Spontaneous vaginal delivery (SVD)	31 (50)	15 (25.4)	**13.455**	**0.003**
Vaginal delivery under epidural analgesia (VD EA)	19 (30.6)	17 (28.8)		
Cesarean section under spinal anesthesia (CS SA)	9 (14.5)	13 (22)		
Cesarean section under general anesthesia (CS GA)	3 (4.8)	14 (23.7)		
Performing an episiotomy^a^	50 (100)	32 (100)		**1**

	Experimental (*n*=63) Mean ±SD	Control (*n*=59) Mean ±SD	*t*	*P*
After birth weight (BMI kg/m^2^)^a^	21.85 ± 2.26	22.73 ± 2.11	2.185	**0.031**
Gain weight at pregnancy^a^	10.70 ± 2.71	13.78 ± 2.48	− 6.407	**0.000**
Gestational week at birth^a^	39.59 ± 0.58	39.32 ± 0.97	1.612	0.611

To vaginal delivery	Experimental (*n*=50) Mean ±SD	Control (*n*=32) Mean ±SD	*t*	*P*
Duration of labor^a^ (hour± minute)	9.46 ± 2.58	15.29 ± 1.77	676.5	**0.000**

^a^Pearson’s chi-square test*,* ^b^*t* test

### A comparison of M-ISI and subgroup scores of the experimental and control groups in the pre-intervention, post-intervention, and postpartum periods

A comparison of Michigan Incontinence Symptom Index (M-ISI) and subgroup scores of the experimental and control groups in the pre-intervention, post-intervention, and postpartum periods is given Table 3. The M-ISI and subgroup SUI and UUI mean scores of the experimental and control groups were similar in the pre-intervention (*t* = 1.887, *p* = 0.905). At the post-intervention, the median score of the experimental group for the M-ISI stress was 2 (min–max = 0–11), while it was 23 (min–max = 14–31) for the control group (*U* = 3.804, *p* < 0.001; Table 2). The median scores of groups for stress incontinence and urge incontinence were statistically significantly different (*U* = 3.804, *p* < 0.001; *U* = 3.610, *p* < 0.001, respectively, Table 2). During the postpartum period, the experimental group showed a median value of 0 (min–max = 0–4) for stress incontinence. In contrast, the control group showed a median value of 7 (min–max = 4–11) for stress incontinence, and there was a statistically significant difference between the two groups (*U* = 3.617, *p* < 0.001; *U* = 3.610, *p* < 0.001, respectively). Similarly, a statistically significant difference was observed between the median values for urge incontinence in both groups (*U* = 3.399, *p* < 0.001).

**Table 3:** Comparison of Michigan incontinence symptom index (M-ISI) and subgroup scores of the experimental and control groups in the pre-intervention, post-intervention, and postpartum periods

Michigan incontinent symptom index (M-ISI)	Experimental group (*n*=63) X̄± SD	Control group (*n*=63) X̄ ± SD	*t*	*P*
Pre-intervention				
Stress incontinence	2.44 ± 2.36	2.79 ± 2.313	− 1539	0.126
Urge incontinence	1.55 ± 1.41	1.90 ± 1.11	− 649	0.518
Total M-ISI	6.06 ± 5.57	13.53 ± 05.46	− 1.887	0.905

Post-intervention^c^	Experimental group (*n*=63) Median (min–max)	Control group (*n*=63) Median (min–max)	*U*	*p*
Stress incontinence	0 (0–3)	9 (4–12)	3.781	**0.000**
Urge incontinence	0 (0–3)	5 (0–7)	3.610	**0.000**
Total M-ISI score	2 (0–11)	23 (14–31)	3.804	**0.000**

Postpartum^c^	*n*=63	*n*=59		
Stress incontinence	0 (0–4)	7 (4–11)	3.617	**0.000**
Urge incontinence	0 (0–5)	4 (0–7)	3.399	**0.000**
Total M-ISI score	1.5 (0–15)	18 (8–28)	3.585	**0.000**

*M-ISI* Michigan incontinent symptom index, *min* minimum, *max* maximum

^a^*t* test, ^b^Pearson’s chi-square test, ^c^Mann–Whitney *U* test

## Discussion

Pelvic floor muscle exercises are a widely used and well-established form of stress incontinence treatment, with success rates ranging from 21 to 84%. [1–3]. This study aimed to determine the effect of pregnancy pilates-assisted childbirth preparation training on childbirth outcomes and urinary incontinence including SUI and UUI during pregnancy and the postpartum period.

To our knowledge, this is the first and only study to evaluate pilates-assisted childbirth preparation training as an antenatal care practice intervention. Pilates exercise intervention for the experimental group started at 28–38 gestational weeks and lasted for 8 weeks. It is known that the incidence of UI increases with the number of gestational weeks [6, 7]. According to this finding, when the pilates exercise-supported birth preparation program is completed after 8 weeks, an increase in the UI scores of pregnant women can be expected. However, in our study, the incontinence scores (UI, SUI, and UUI) of pregnant women who did pilates exercise were found to be significantly lower than those of women who did not do pilates exercise. Additionally, women in the experimental group had significantly lower urinary incontinence frequency and volume and perceived stress urinary incontinence severity score after participation than the control group. All these findings of our study show that pilates exercise is effective in reducing urinary incontinence during pregnancy.

In our study, 8 weeks of pilates exercise-assisted childbirth preparation training were monitored, together with functional parameters such as weight gain at pregnancy (kg) and postpartum, its effects on childbirth parameters such as delivery method, analgesia, duration of labor and newborn weight and episiotomy. However, 8 weeks of pilates exercise had no effect on newborn weight and episiotomy. In the findings of the randomized-controlled study conducted by Rodríguez-Díaz et al. [25], with 105 pregnant women, where an 8-week pilates training intervention was applied, the effects of parameters similar to our study were observed. Rodríguez-Díaz et al. [25] reported that at the end of 8 weeks, positive improvements were achieved in all parameters, including newborn weight and episiotomy, in contrast to the findings of our study. From another perspective, the results of the episiotomy were inconsistent with those of Rodríguez’s study [25] and Ghandali’s study [18]. This is because episiotomy is routinely performed on almost all primiparous mothers in Turkey. Therefore, we did not expect any change between the groups in our study.

In our study, the pilates exercise during pregnancy reduced the duration of labor. In line with our study, in the previous studies, the duration of labor in the experimental group was shorter than that in the control group [26, 27]. Previous studies were conducted by Gahandi et al. and Barakat et al., which also reported shorter labor duration in the experimental group compared to the control group [18, 26]. Proper exhalation techniques and diaphragmatic breathing can strengthen the diaphragm and assist the uterine muscles during labor and childbirth, resulting in a smoother delivery process [14, 18]. During the second stage of childbirth, the infant’s head gradually descends through the completely dilated cervix. This process is facilitated by the force of uterine contractions and intrauterine pressure. However, if this pressure persists for an extended duration, it can result in ischemic necrosis of the pelvic tissues, including nerves and muscles. This can lead to lasting damage to the pelvic floor, causing stretch injuries and neuromuscular damage. Therefore, a prolonged second stage of labor can elevate the likelihood of soft-tissue injury and long-term damage to the pelvic floor. In this study, regular pilates exercise has been shown to lower the risk of long-term damage and injury to the pelvic muscles. This, in turn, contributes to reducing the incidence of Stress Urinary Incontinence (SUI) and Urge Urinary Incontinence (UUI) in the postpartum period.

In our study, results showed that almost all of the births of women who exercised during pregnancy resulted in vaginal birth, while slightly more than half of the women in the control group gave birth vaginally. The vaginal birth rate of the control group of our study is similar to the results (54.4%) of the Turkish Demographic and Health Survey (TDHS) [28]. The vaginal birth rate of the experimental group was much higher than the TDHS results [28]. However, it was as recommended by the World Health Organization (WHO) [29]. Studies by Rodríguez et al. [25], and Sarpkaya Güder et al. [13] demonstrated a statistically significant difference in the mode of delivery between participants who had pilates exercise intervention and the control groups, with the rate of vaginal deliveries being higher in the pilates group. However, there is a result which was different, from the results of a previous study conducted in Northern Cyprus examining the effect of pregnancy pilates-assisted childbirth preparation training on childbirth fear and neonatal outcomes [14], and one conducted in Iran [18]. The first study was conducted in Northern Cyprus, and here, the vaginal birth rate of the experimental group was not statistically higher than the control group. Sarpkaya Güder et al. [14] emphasized in their study that this was due to the high rate of cesarean births in Northern Cyprus, which is also reflected in the study results. In addition, the other study, which examined the birth outcomes of pilates intervention applied during pregnancy in Iranian women, is different from our results. In this study, 88.2% of the women in the intervention group and 80.8% of the women in the control group had a vaginal birth. There was no statistically significant difference between the two groups in terms of delivery method [14]. In our study, it is thought that education components as well as pilates exercise are effective in the fact that the rate of vaginal birth is significantly higher than cesarean birth.

Numerous studies have shown that age, parity, infant birth weight, BMI, vaginal delivery, and episiotomy are risk factors on postpartum UI [4, 7, 11, 16, 17, 30]. However, cesarean delivery, especially elective cesarean, is known to protect against postpartum UI [31]. According to the findings of our study, although the women in the experimental and control groups were statistically similar in terms of age, parity, baby birth weight, and episiotomy, the severity and incidence of postpartum UUI and SUI were lower in the women in the experimental group who did pilates exercise.

BMI and high-impact exercising were found to be the main associated risk factors [10]. The evidence from Systematic Review and Meta-analysis reported that maternal BMI might be a risk factor for postpartum SUI, and that women with higher maternal BMI would have a higher risk [5]. Vissers et al. [32] reported that after a 6-month intensive weight loss program, there was a 47% decrease in the frequency of urinary incontinence with an average weight loss of 8%. In our study, it is thought that the lower BMI of the women who did the pilates exercise compared to the control group who did not do the pilates exercise may be effective in the statistically significant decrease in urinary incontinence and stress incontinence in the postpartum period.

### Strengths and limitations

Conducting a randomized-controlled trial using a pregnancy pilates intervention allowed us to show a comprehensive picture of reducing the severity of UI symptoms of the onset of pregnancy and the early postpartum provides a good basis for the development of women-centered interventions and for further research. One of the strengths of our study is that it is a randomized-controlled and single-blind study. However, our study results have limitations. First of all, they only represent the results of nulliparous women. The research results are limited to urinary incontinence results in the postpartum period 1 month after birth. The factors that influence postpartum UI in the long term should be investigated. Additional research is needed to study the effects of exercise on pregnancy-specific conditions and outcomes and to clarify further effective behavioral counseling methods and the optimal type, frequency, and intensity of exercise.

### Clinical implications

A moderate-intensity prenatal pilates exercise program reduces the severity of UI symptoms during pregnancy and the early postpartum period. It contributes to reducing the risk of UI including SUI and UUI in the early postpartum period. Pilates exercise-assisted childbirth preparation training was monitored, together with functional parameters such as weight gain at pregnancy (kg) and postpartum, and its effects on childbirth parameters, such as delivery method, analgesia, duration of labor, and newborn weight.

## Conclusions

An 8-week childbirth preparation training program assisted by pregnancy pilates (2 days a week, 1 h a day theoretical, 45 min pregnancy pilates, and 15 min breathing exercise) was effective in reducing the severity of UI including UUI and SUI. Pilates exercise during pregnancy contributed to the reduction of stress incontinence and urge incontinence problems both during pregnancy and in the postpartum period. A pilates exercise-assisted childbirth preparation training program helped weight control by contributing to less weight gain during pregnancy, and it was also effective in increasing the rate of vaginal births and shortening the birth time. It is recommended that nurses, midwives, and other specialist healthcare professionals who provide birth preparation training integrate pilates exercise into their programs, as well as breathing and relaxation exercises.

## Data Availability

The data are available from the corresponding author upon reasonable request, provided that the necessary legal recourse is made.
