# Correction: The effect of pregnancy pilates-assisted childbirth preparation training on urinary incontinence and birth outcomes: a randomized-controlled study

**DOI:** 10.1007/s00404-024-07822-6

**Published:** 2024-11-15

**Authors:** Gonca Buran, Serap Erim Avcı

**Affiliations:** 1 Department of Obstetrics and Gynecology Nursing, Faculty of Health Sciences, Bursa Uludag University, 16059 Nilüfer, Bursa Turkey; 2 Gynecology and Obstetrics, Pregnancy Education and Counseling Center, Nilüfer, Bursa Turkey

**Correction: Archives of Gynecology and Obstetrics (2024) 310:2725–2735** 10.1007/s00404-024-07653-5

In this article, the second author’s name Serap Erim Avcı was incorrectly written as Serap Erim.

Secondly, under discussion section on page 9, the reference was incorrectly mentioned as 14 and should have been 31.

The original article has been corrected.

